# Alternative ‘block and delivery’ approach for alcohol septal ablation in hypertrophic cardiomyopathy with ischemia: a case report

**DOI:** 10.1186/s43044-025-00636-0

**Published:** 2025-04-28

**Authors:** S. M. Mamun Iqbal, Neuton Mondal, Mafuza Tabassum Khan, Ashir Faishal Hamim, Nurullah Mujahid Majumder, Anindita Das Barshan, Mohammad Jahid Hasan

**Affiliations:** 1 M H Samorita Hospital and Medical College, Dhaka, Bangladesh; 2 Pi Research and Development Centre, Dhaka, Bangladesh; 3 Tropical Disease and Health Research Center, Dhaka, Bangladesh

**Keywords:** Hypertrophic cardiomyopathy, Coronary artery disease, ‘Block and delivery’, Alcohol septal ablation

## Abstract

**Background:**

Hypertrophic cardiomyopathy (HCM) is a prevalent hereditary cardiac disorder characterized by marked myocardial hypertrophy, which may lead to impaired diastolic function and relative myocardial ischemia. On rare occasions, HCM coexists with coronary artery disease (CAD), complicating therapeutic decisions due to heightened risks of heart failure and ischemic events. Treatment options for these patients commonly include surgical myomectomy or alcohol septal ablation, traditionally performed using an over-the-wire (OTW) balloon catheter. Here, we present a case in which a modified 'block and delivery' alcohol septal ablation technique was utilized, instead of the conventional OTW approach, in a patient with concurrent HCM and CAD within a resource-limited setting.

**Case presentation:**

A 40-year-old Asian female presented with angina and acute heart failure in our clinic. Diagnostic evaluations revealed hypertrophic obstructive cardiomyopathy (HOCM) with severe left ventricular outflow tract obstruction (LVOTO) and significant coronary artery stenosis. Due to equipment constraints, the patient underwent staged interventions: a percutaneous coronary intervention (PCI) followed by alcohol septal ablation using a modified technique. This intervention effectively reduced the LVOT gradient from 108 to 17 mmHg. At the one-year follow-up, the patient demonstrated good health, complete symptom resolution, and a normal left ventricular outflow tract gradient.

**Conclusion:**

This case illustrates the feasibility of employing a modified alcohol septal ablation technique in resource-limited settings, highlighting the importance of adaptable and innovative approaches in managing complex cardiac conditions.

## Background

Hypertrophic cardiomyopathy (HCM) is a genetic cardiac disorder characterized by thickening of the heart muscle, notably the interventricular septum, which results in left ventricular outflow tract obstruction (LVOTO) and compromised cardiac function. HCM is among the leading causes of sudden cardiac death in young athletes, with an incidence of approximately 1 in 1,000 person-years [1]. While HCM primarily affects cardiac muscle, its association with coronary artery disease (CAD) presents a significant clinical challenge. Though the coexistence of CAD and HCM is infrequently documented, it markedly heightens the risk of myocardial ischemia and adverse cardiac events [2]. Multiple factors may contribute to the development of CAD in patients with HCM. Myocardial thickening can lead to reduced blood supply, increased myocardial oxygen demand, and heightened microvascular dysfunction [3]. Over time, these factors can precipitate ischemia, even in the absence of traditional coronary artery obstructions. Prompt diagnosis of CAD in HCM patients is therefore essential to optimize therapeutic strategies and improve both short-term and long-term clinical outcomes. Untreated ischemic episodes in these patients may further deteriorate cardiac function and elevate mortality risk [4].

This case report discusses a 40-year-old female patient with hypertrophic cardiomyopathy and coexistent coronary artery disease, who presented with worsening angina and dyspnea. Following coronary angiography, a two-stage treatment plan was developed, comprising percutaneous coronary intervention (PCI) followed by alcohol septal ablation. Due to the unavailability of the conventional OTW catheter during the procedure, an alternative 'block and delivery' technique was employed for the alcohol septal ablation, highlighting the procedural complexities of managing HCM in resource-limited settings such as Bangladesh.

*Case Presentation* A 40-year-old female presented with severe, non-radiating central chest pain and progressive dyspnea persisting for one day. Her chest pain was described as compressive and continuous, initially without radiation, and later accompanied by exertional dyspnea. She reported a history of similar exertional dyspnea over the past three years, with a recent exacerbation of symptoms. Her medical history was notable for hypertension, bronchial asthma, and four episodes of cardiac syncope occurring during moderate physical exertion over the last eight years.

On examination, the patient appeared dyspneic and was maintaining a propped-up position. Vital signs indicated a heart rate of 110 beats per minute, blood pressure of 110/60 mmHg, and a respiratory rate of 26 breaths per minute. Jugular venous pressure (JVP) was raised, measuring approximately 8 cm above the sternal angle.

Precordial examination revealed a forceful, sustained apex beat. The first heart sound (S1) was soft, while the second heart sound (S2) was normal. A pansystolic murmur was audible at the apex with radiation toward the axilla. Additionally, a non-radiating ejection systolic murmur was detected at the left parasternal edge. Respiratory examination indicated bilateral vesicular breath sounds with prolonged expiration, diffused rhonchi, and inspiratory crepitations in the lower zones of both lungs.

**Fig. 1:**
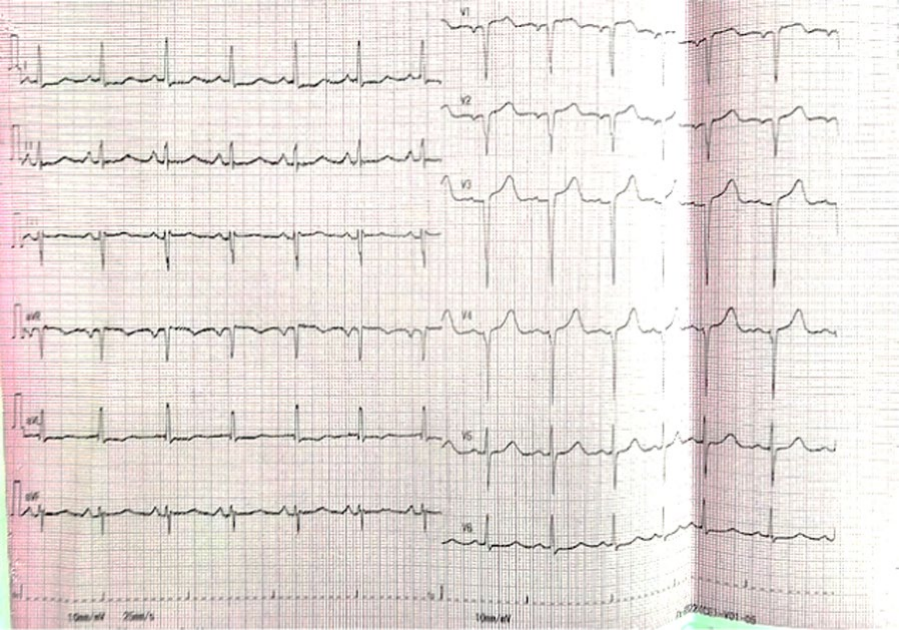
Pre-procedure ECG

Initial laboratory tests revealed negative results for Troponin-I on two consecutive samples taken three hours apart, along with elevated NT-proBNP levels. The pre-procedure electrocardiogram demonstrated left atrial enlargement and poor R wave progression (Fig. 1). Chest X-ray findings were consistent with cardiomegaly. Transthoracic echocardiography confirmed hypertrophic cardiomyopathy (HCM) with systolic anterior motion of the anterior mitral leaflet, a severe left ventricular outflow tract (LVOT) gradient measuring 132 mmHg, and moderate mitral regurgitation. The septal thickness was measured at 20 mm (2.0 cm)**.**

Considering the progressive symptomatology, coronary angiography was performed. The angiogram revealed 90% stenosis in the posterior descending artery (PDA) branch of the right coronary artery (RCA) and 40–50% stenosis in the proximal left anterior descending (LAD) artery, with a myocardial bridge identified in the mid-LAD.

## Therapeutic approach

Initially, the patient was treated for acute heart failure with intravenous furosemide, oral spironolactone, and ivabradine. A two-stage interventional approach was subsequently planned, involving percutaneous coronary intervention (PCI) in the posterior descending artery (PDA) and alcohol septal ablation, with an interval of 4–6 weeks between procedures. In the first stage, PCI was performed on the PDA with deployment of a 2.5 × 23 mm drug-eluting stent. Following PCI, the patient was prescribed prasugrel 10 mg od, aspirin 75 mg od, rosuvastatin 10 mg od, and glyceryl trinitrate 2.6 mg bd, in addition to her heart failure medications. Her condition showed gradual improvement, allowing for discharge four days post-procedure.

However, the patient was readmitted 14 days later, presenting with severe rest angina and dyspnea. A repeat coronary angiogram (CAG) confirmed patency of the PDA stent, leading to the decision to proceed with urgent alcohol septal ablation. This procedure presented several challenges: the patient’s condition was rapidly deteriorating due to acute heart failure, and the conventional over-the-wire (OTW) balloon catheter was unavailable due to post-pandemic supply issues. Consequently, the cardiac team opted to perform an alternative 'block and delivery' technique, employing a simultaneous balloon and microcatheter in place of the OTW balloon. An additional challenge was counseling the patient’s family. After a comprehensive discussion detailing the risks and benefits of the proposed alternative approach, as well as the option of surgical myomectomy, the patient and her family elected to proceed with the recommended alternative treatment.

**Fig. 2:**
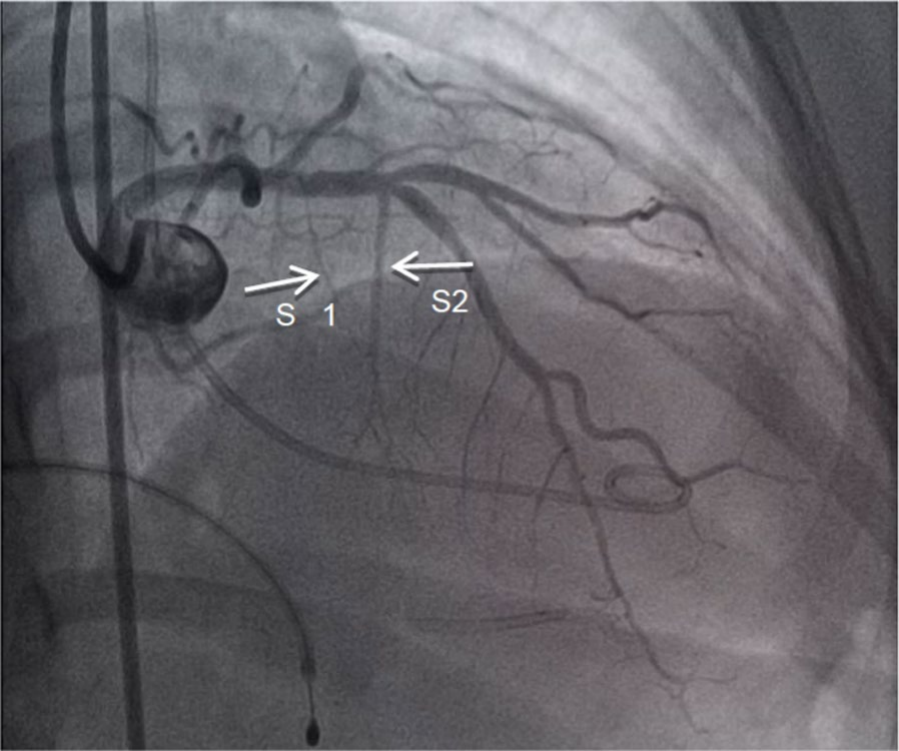
First septal branch (S1 with an arrow) and second septal branch (S2 with an arrow)

The procedure commenced with temporary pacing via the femoral vein, while a 5Fr Pigtail catheter was introduced through the femoral artery to assess the baseline left ventricular outflow tract (LVOT) gradient, recorded at 108 mmHg. A JL 6Fr guiding catheter was used to engage the left coronary artery (LCA), followed by angiography. The second septal artery was identified as the primary septal branch supplying the mid-basal region of the interventricular septum, as confirmed by echocardiographic contrast injection (Fig. 2).

**Fig. 3:**
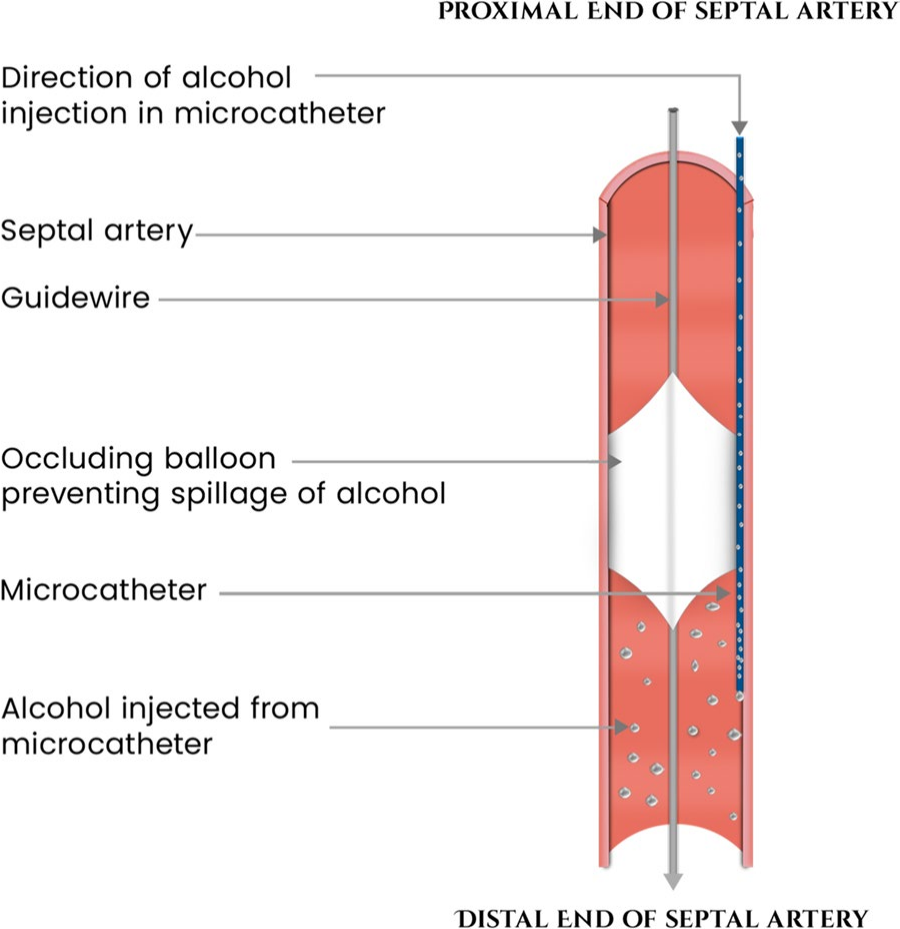
Diagram of balloon occlusion technique for alcohol septal ablation

**Fig. 4:**
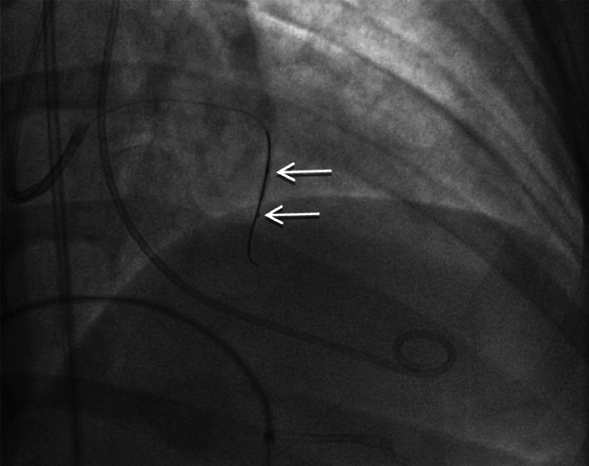
Second septal branch: (upper arrow) shows inflated balloon and (lower arrow) shows tip of the microcatheter

A Fine Cross Terumo micro-guide catheter 0.014′′ 180 cm was advanced over a Run-through wire into the second septal branch. The wire was temporarily removed and then reintroduced into the septal branch alongside the micro-guide catheter. A 2.0 × 12 mm non-compliant balloon was positioned over the wire within the septal artery, with its distal end situated 5–10 mm proximal to the tip of the micro-guide catheter. The balloon was inflated to 12 ATM, and angiography was performed in two stages. Initially, contrast was administered via the guiding catheter to confirm occlusion of the septal artery, followed by selective angiography through the micro-guide catheter to verify the absence of contrast reflux into the left anterior descending (LAD) artery or collateral vessels. With the balloon still inflated, 2 ml of 95% ethyl alcohol was gradually infused over two minutes through the micro-guide catheter, followed by a 0.3 ml saline flush (Fig. 3). The balloon remained inflated for 10 min to complete the ablation (Fig. 4).

Upon completion, both the micro-guide catheter and balloon were removed. Post-procedural angiography confirmed complete occlusion of the distal segment of the second septal branch. A transient reduction in blood flow (TIMI-2) was observed in the LAD, which improved following intracoronary administration of glyceryl trinitrate (GTN). Despite successful occlusion of the second septal artery, the LVOT gradient remained unchanged. Attention was then directed toward locating an alternative septal branch with therapeutic potential. The first septal branch, though smaller in size and length, showed a significant reduction in the LVOT gradient upon balloon occlusion. Therefore, alcohol ablation of the first septal branch was also performed using the same technique, guided by contrast echocardiographic findings.

## Outcome

**Fig. 5:**
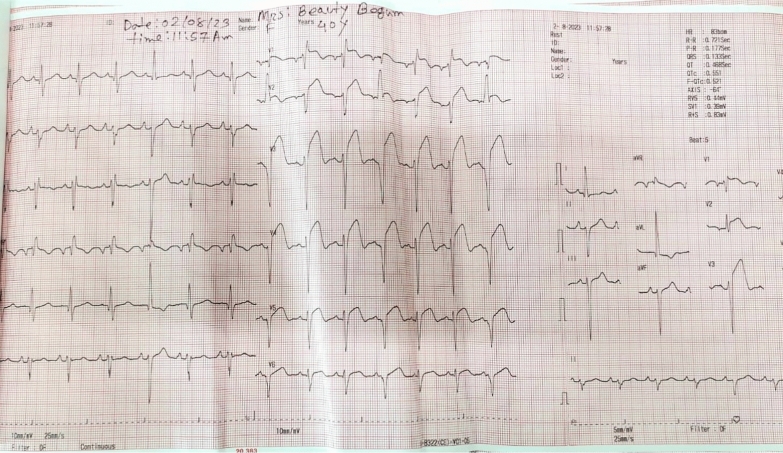
Post-procedure ECG demonstrating septal infarction

Post-procedural assessment revealed a marked reduction in LVOT gradient from 108 to 17 mmHg, as confirmed by transthoracic echocardiography. The patient remained hemodynamically stable throughout the procedure, with no significant complications. Post-procedure ECG revealed ST elevation in Lead V1–V2 demonstrating septal infarction (Fig. 5). The temporary pacemaker was removed after 48 h of observation, as no evidence of AV block was detected. The patient was discharged five days post-procedure and showed excellent recovery. At the one-year follow-up, the patient remained symptom-free, with a normal LVOT gradient on echocardiogram.

## Discussion

The management of patients with coexisting hypertrophic cardiomyopathy (HCM) and coronary artery disease (CAD) presents distinctive therapeutic challenges, especially in resource-limited settings. This case underscores several critical clinical and interventional issues that merit further exploration.

The coexistence of HCM and CAD is relatively rare, first documented in 1973 [5]. Our patient developed CAD at an unusually young age (40 years), which is earlier than in typical CAD patients without HCM. Previous studies have highlighted a high prevalence of acute coronary syndrome with non-obstructive coronary artery disease (ACS-NOCA) in patients with HCM [6]. Significant coronary artery disease has been reported in only 14–19% of HCM patients [7] though some research suggests CAD prevalence may be comparable in both HCM patients and controls [8]. Diagnosing CAD in HCM patients is challenging due to overlapping symptoms. In this case, the presentation of acute heart failure emphasizes the need for a high index of suspicion and comprehensive coronary evaluation in patients with HCM.

Our case also highlights the importance of personalized treatment strategies, particularly in situations where conventional treatment guidelines don’t fit. As the patient presented with acute heart failure, surgical myomectomy could not be performed. The management of such patients aligns with the previous evidence supporting alcohol septal ablation for individuals such deteriorating symptoms although surgical myomectomy is still the gold standard [9]. The clinical improvement of the patient, as demonstrated by the LVOT gradient’s decrease and subsequent stabilization, supports the efficacy of this strategy in situations that are carefully chosen.

Our two-step approach, performing PCI followed by septal ablation, is unique, as few reports have documented such combination therapies, and the optimal timing for combined interventions remains unclear. Prior case studies indicate that primary PCI can significantly reduce LVOT gradient and improve left ventricular function in HCM patients [10]. However, in our case, PCI alone did not alleviate symptoms, necessitating subsequent septal ablation.

One of the most notable aspects of this case is the implementation of an alternative 'block and deliver' approach for alcohol septal ablation in the absence of conventional over-the-wire (OTW) catheters. This modified technique, traditionally used in distal coronary artery perforation management, offers an innovative solution for resource-limited settings [11]. By utilizing commonly available equipment, such as a standard balloon catheter and microcatheters, this technique may be more feasible in developing countries. Our procedural success, evidenced by the reduction in LVOT gradient from 108 to 17 mmHg, aligns with outcomes from conventional septal ablation series. The sustained improvement at the one-year follow-up indicates that this modification can produce outcomes comparable to traditional methods.

This case is particularly relevant to healthcare delivery in developing countries such as Bangladesh, where access to specialized cardiac equipment may be limited and financial constraints often impact treatment decisions. Our success illustrates that with appropriate patient selection and skilled adaptation, complex cardiac procedures can be modified to meet resource limitations without compromising efficacy. This highlights the critical need for innovative adaptations of conventional methods in such settings, and our experience suggests that these modifications can be safely and effectively executed.

Looking forward, this case raises important questions about the long-term outcomes of modified 'block and deliver' septal ablation techniques and the optimal timing for interventions in patients with both HCM and CAD. Standardized protocols for managing these complex cases in resource-limited environments are essential. Future research should focus on validating these modified techniques through larger studies and establishing clear guidelines for their application in various healthcare settings.

## Conclusion

This case highlights several crucial aspects of managing complex cardiac conditions in resource-limited settings. It illustrates that alternative procedural adaptations to address equipment limitations can be implemented effectively without compromising therapeutic efficacy. The positive outcome of our staged approach in managing concurrent hypertrophic cardiomyopathy (HCM) and coronary artery disease (CAD) provides evidence that complex cardiac interventions can be successfully executed with modified techniques, given careful patient selection and sufficient technical expertise. This experience supports the feasibility of tailored interventions in settings where specialized resources are constrained.

## Data Availability

All data were available to the lead author and could be found upon the reasonable request to the corresponding author.

## Abbreviations

CAD: Coronary artery disease
CAG: Coronary angiogram
GTN: Glyceryl trinitrate
HCM: Hypertrophic cardiomyopathy
JVP: Jugular venous pressure
LAD: Left anterior descending
LCA: Left coronary artery
LVOTO: Left ventricular outflow tract obstruction
OTW: Over the wire
PCI: Percutaneous coronary intervention
PDA: Posterior descending artery
RCA: Right coronary artery
